# Epidemic malaria and warmer temperatures in recent decades in an East African highland

**DOI:** 10.1098/rspb.2010.2020

**Published:** 2010-11-10

**Authors:** David Alonso, Menno J. Bouma, Mercedes Pascual

**Affiliations:** 1 Community and Conservation Ecology Group, University of Groningen, CEES, Haren, The Netherlands; 2 London School of Hygiene and Tropical Medicine, London, UK; 3 Department of Ecology and Evolutionary Biology and Howard Hughes Medical Institute, University of Michigan, Ann Arbor, MI, USA

**Keywords:** epidemic malaria, climate change, highland malaria, malaria population model, temperature trend

## Abstract

Climate change impacts on malaria are typically assessed with scenarios for the long-term future. Here we focus instead on the recent past (1970–2003) to address whether warmer temperatures have already increased the incidence of malaria in a highland region of East Africa. Our analyses rely on a new coupled mosquito–human model of malaria, which we use to compare projected disease levels with and without the observed temperature trend. Predicted malaria cases exhibit a highly nonlinear response to warming, with a significant increase from the 1970s to the 1990s, although typical epidemic sizes are below those observed. These findings suggest that climate change has already played an important role in the exacerbation of malaria in this region. As the observed changes in malaria are even larger than those predicted by our model, other factors previously suggested to explain all of the increase in malaria may be enhancing the impact of climate change.

## 1. Introduction

The impact of climate change on vector-transmitted infectious diseases is a pressing and still highly debated question, mostly addressed with scenarios for the future. We are now in a position, however, to also consider the recent past and address whether climate change has already influenced the temporal population dynamics of infectious diseases in recent decades when the first significant signs of a change in climate have been detected. This question is particularly relevant for regions known to be potentially sensitive to climate change. In the case of malaria, these regions lie at the edge of its geographical distribution, in highlands and desert fringes, where temperature or rainfall limits the development of the parasite and the abundance of the mosquito vector [1,2]. In these transition regions, where transmission is low and intermittent, the population dynamics of malaria are formally described as ‘unstable’ or ‘epidemic’, exhibiting intermittent seasonal outbreaks with high mortality because of low levels of immunity in the population. Questions about the effect of warmer temperatures are especially relevant in highland regions because of the clear relationship between altitude and temperature, and the known influences of temperature on both the *Plasmodium* parasite and the *Anopheles* vectors. Temperature influences the development of the parasite within the mosquito in a nonlinear manner [3]—the so-called extrinsic incubation period, also known as sporogony, which must be completed within the lifetime of the vector for transmission to be possible. In between the two lethal extremes, higher temperatures also speed up the rate of development of *Anopheles* mosquitoes and shorten the gonotrophic cycle, which can increase the biting rate [4]. Our retrospective analysis of local temporal dynamics complements broad-scale scenarios for the impact of climate in decades to come, primarily focused on global risk maps for future geographical distributions of malaria (and other vector-borne diseases) [5–10].

Although several highland regions in East Africa have already experienced a significant exacerbation in the size of malaria outbreaks over the last three decades (see [11–13] and references therein), the role of temperature patterns remains controversial [14]. No retrospective study has evaluated the effect of local warming trends with a quantitative approach that explicitly considers the full transmission cycle of malaria [11,13,15–18]. The re-analyses of the temperature time series for the global gridded climate product known as Climate Research Unit (CRU) [19] has shown that statistically significant trends are present in these regions and that these could generate significant increases in vector abundance [17]. The relevance of the gridded temperature records has been questioned for a landscape of rapid altitudinal change for which spatial averages can poorly represent local temperatures at the coarse spatial resolution of the grid (0.5° per 0.5° lat. × long.) [18]. A graphical comparison of the temperature records for the CRU grid point and the local stations in a Kenyan highland has shown, however, that both time series exhibit a similar increasing trend, with lower overall (and also mean) values for the latter, as the local measurements can better represent a specific (in this case, higher) altitude [20].

We consider here both disease transmission and local temperatures in a highland region of Western Kenya for which a time series of confirmed malaria cases is available monthly for over three decades. A malaria population model that couples the dynamics of the disease in both the mosquito vector and the human host is used to evaluate the impact of a trend in local temperatures on the population dynamics of the disease. The malaria model is parametrized using the hospital records for the 1970s and beginning of the 1980s, a period of low incidence preceding epidemic behaviour. The expected dynamics of cases are then projected forward for the following decades with and without the temperature trend. This approach differs by design from one based on fitting the complete set of data, and therefore from one that would seek to explain *a priori* the full increase in cases with the temperature trend. This allows us to quantify the effect of warming without precluding the change of other major drivers, such as drug resistance and human population movement, also acting on malaria across these decades. Comparisons of the projections with and without the temperature trend allow us to address how much of a change in the size of epidemics the observed warmer temperatures could have generated. Our findings support a significant effect of warmer temperatures on the exacerbation of malaria in this East African highland, while also allowing for a role of other factors. These conclusions are robust to different model structures for the population dynamics of the disease and to other variations in our analyses. We end with limitations of the analyses, discussion on questions of population growth and open areas for the future.

## 2. Data

**Figure 1.**
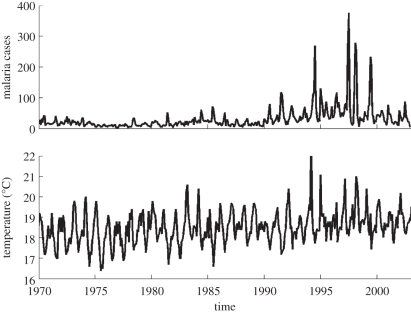
Monthly time series for (*a*) malaria cases and (*b*) mean temperatures. The malaria data consist of confirmed cases for inpatients from the admission records for 1966–2002 at the hospital serving a tea plantation composed of several estates (Brooke Bond Farms, now Unilever Tea Kenya Ltd; latitude 0.3° S, longitude 35.37° E, elevation 1780–2225 m) [15]. The temperature data were obtained by dovetailing the records from two meteorological stations within the tea estates, together with adjustments for altitude based on mean temperature data from a number of stations in Kenya spanning a broader altitude range (see §2 and electronic supplementary material, figure S1 and §5 for details). The data from the Tea Research Foundation (TRF) meteorological station were used up to 1992, and from December 1997 to March 1998 when the second record was missing; the data from the official meteorological station were used from 1992 onwards by adjusting these to the altitude of the TRF time series. The graph shows the temperature time series adjusted for 1780 m.

The data consist of a monthly time series of malaria cases reported from hospital records in the Kericho district of the Kenyan highlands, on the western side of the Great Rift Valley near Lake Victoria (figure 1*a*; see caption for details). We consider a local temperature time series generated by dovetailing the records from two local meteorological stations adjacent to the tea plantation (figure 1*b*; see caption and §5 in the electronic supplementary material for details) [20].

The reported population size for workers and their dependants in the tea estates ranges from 50 000 to 100 000 in the literature [15,16]. We focus on the lower half of the altitude range (from 1780 to 1980 m), where the impact of warming would be expected to be most evident, and consider first a total constant population of 50 000. We then address the sensitivity of our results to this number, as well as the possible effect of an increasing population outside the tea estate (electronic supplementary material, figure S12 and §11.2) [21]. We present results for altitudes of 1780 and 1880 m (the lower boundary and middle of this range, respectively). Figure 1*b* shows the temperature time series adjusted for 1780 m. There is a clear trend in the data of an increase of approximately 1°C over 30 years. Our goal is to evaluate the possible impact of this observed pattern on the population dynamics of malaria using a coupled human–mosquito transmission model.

The mosquito component of our epidemiological model (electronic supplementary material) requires rainfall as a second climate driver. We use two rainfall datasets in our analyses. The first one is a monthly rainfall time series from a local meteorological station in the Kericho district from 1970 to 2003 (Kericho Chagaik Estate, 0°20′ S, 35°20′ E, 6000 ft; fig. 3 in [22]). We subdivided the monthly cumulative rainfall equally into daily values to estimate the amount of rainfall per day, since all our rates in the model equations are expressed per day. To take into account the autocorrelation patterns resulting from daily variability, we also considered a rainfall time series consisting of daily values from another meteorological station in the district from 1973 to 2003 (Hail Station, 0°22′ S, 35°16′ E, 6480 ft; fig. S1 in [22]). The treatment of missing data is described in the electronic supplementary material.

## 3. Results

**Figure 2.**
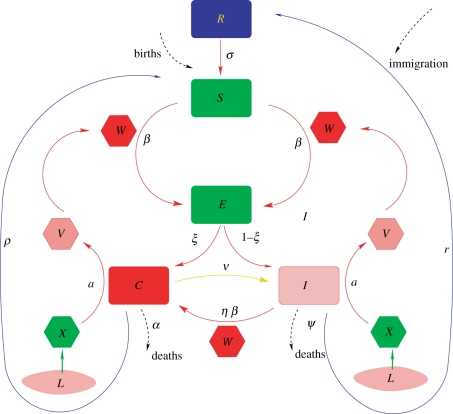
Coupled mosquito–human model of malaria dynamics. The population is subdivided into a number of classes. In particular, two types of infected individual are considered: those who present symptoms and therefore receive some sort of clinical treatment (*C*), and those who acquire asymptomatic infection (*I*) but are nevertheless infectious and can transmit the parasite to the vector. A recovered class (*R*) consists of individuals who have cleared parasitaemia, or have too low a level of parasitaemia to effectively infect the vector. The replenishment of susceptibles through immigration or births (*B*) and individual losses owing to mortality—or more generally, population turnover (*δ*)—are considered to balance each other so that the total population *N* remains constant. A constant maximum size of the worker population and their dependants in the tea estates supports this assumption [15]. The coupling to the mosquito component of the model occurs through the ‘force of infection’ (*β*), the *per capita* rate at which susceptible individuals become infected. This rate contains two terms to allow for two different sources of infection, for local and external transmission, respectively (electronic supplementary material). The local force of infection depends on the number of infected mosquitoes *W*, and the mosquito population is subdivided into larvae (*L*), and adults, with uninfected adults (*X*) becoming exposed (*V*) when they bite an infectious human (electronic supplementary material). Only a fraction of infections in humans (*ξ*) fully develops severe malaria symptoms and then receives clinical treatment (*C*). Asymptomatic but infectious individuals (*I*) can present a relapse of severe malaria symptoms if they are bitten again, but the *per capita* transmission rate (*β*) of this process is decreased by a factor *η*. The clearance or recovery rate for treated infected and sick (*C*) and non-treated infected individuals (*I*) are *ρ* and *r*, respectively. Recovered individuals in *R* lose immunity at rate *σ* and return to *S* with a relaxation time that depends on mosquito exposure (see electronic supplementary material for details).

A malaria population model that couples the dynamics of the disease in both the mosquito vector and the human host allows us to evaluate the potential response to the temperature trend. The variables and flows in the model are illustrated in figure 2 and described in the electronic supplementary material together with variants of model structure, including a different representation of immunity. The model can be seen as a dynamic map from the climate time series of temperature and rainfall to the disease variables of interest over time, including the numbers of cases, adult mosquitoes and infections in both humans and vectors. The results described here correspond to a large number of model simulations that compare the predicted dynamics after 1985 in the absence and presence of the temperature trend (electronic supplementary material). The fitting of the model to the data up to 1985, using a genetic algorithm to maximize its likelihood (electronic supplementary material), provides an ensemble of best parameter sets that reflects the uncertainty in parameter estimation. The average values of the fitted parameters and their uncertainty are reported in electronic supplementary material, table S1.

Repeated simulations using these parameter sets generate a distribution of predicted malaria cases for any given month and for two temperature regimes: the observed pattern and the baseline, with no trend, generated by sampling years from the 1970s at random (electronic supplementary material). The latter time series mimics the no-trend scenario in which temperature values in the 1990s would have been ‘like’ those in the 1970s (except for interannual patterns of autocorrelation). We start with the results for an altitude of 1780 m and the monthly rainfall data.

**Figure 3.**
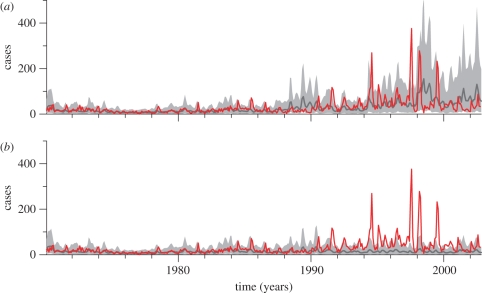
Numerical simulations of the model (*a*) with and (*b*) without the trend in temperature. The numerical simulations generate a distribution of cases for each month reflecting the uncertainty in parameter values of the model (electronic supplementary material). There are three sources of uncertainty in these simulations. First, our parameter search produces a family of solutions. We use this whole family of parameter sets to simulate the model repeatedly. Second, each simulation without the trend considers a different random sample of temperatures in the 1970s. Third, the model includes an error model to account for uncontrolled, unavoidable variability from processes not explicitly modelled in our deterministic approach. Thus, the resulting simulations give us a distribution of cases (as well as infected numbers) for each month. We plot here the median number of cases (50% percentile, dark grey line), together with the range from the 5 to 95% percentiles (light grey shading) of the distribution of predicted cases for each month. Comparison of (*a*) with (*b*) shows the effect of warmer temperatures on the dynamics of the malaria model. The observed cases are plotted in red.

**Figure 4.**
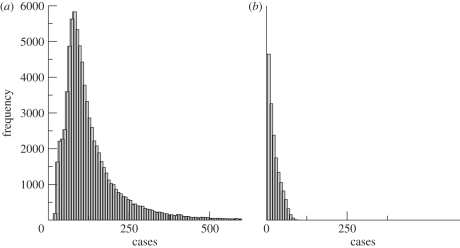
Histograms of predicted cases at the seasonal peaks for the model (*a*) with and (*b*) without the trend in temperatures.

With the observed temperatures, the simulations generate a significant increase in the size of epidemics from the 1970s to the 1990s. Figure 3 compares the projected dynamics of cases in the presence and absence of the temperature trend. For each month, we have illustrated the expected number of cases, superimposed on the 5 and 95 per cent percentiles of the case distribution. The temperature trend produces an increase in cases that is clearly absent in the baseline scenario of no trend. The distribution of simulated cases in the 1990s (and particularly during epidemics) is highly asymmetric, exhibiting a long tail (figure 4). This implies a large range of plausible values above the median, including extreme peaks, with low probability. Warmer temperatures shift the distribution of the seasonal peaks towards much larger values, typically eight times larger than those of the simulations without the trend, if we compare their most likely values (figure 4).

Comparison of the simulated and observed cases (figure 3*a*) shows that the size of observed epidemics falls typically within the uncertainty of the projections. However, the median of the simulated cases is typically below the observed values, and simulated cases tend to anticipate the timing of the large peaks in 1997–1998 (we return to discussion of these two differences below).

When the daily rainfall time series is considered, results show a comparable increase in cases in the presence versus the absence of a temperature trend. The above results are also robust to changes in model structure. When the treated cases enter the recovered (immune) class instead of the susceptible class, very similar dynamics and effects of temperature are obtained (see electronic supplementary material, table  S2, for estimated parameters). A more significant change of model structure that considers a different representation of population immunity [23] predicts again a significant effect of warming (electronic supplementary material, figure S3 and table S3). When the three models are considered at the same altitude and run with the same rainfall, the estimated parameters are remarkably similar, as are the projections for both the malaria cases (electronic supplementary material, figure S5) and other epidemiological quantities, such as the entomological inoculation rate (EIR; electronic supplementary material, figure S6) and vectorial capacity (not shown). Similar results on the response to temperature were also obtained for an altitude of 1880 m. However, the likelihood of the model was higher for the lower altitude of 1780 m (electronic supplementary material), consistent with an effect on transmission that is felt most strongly in the lower part of the tea estate.

To better understand the response of cases to warming, we consider how the equilibrium of the model (with no seasonality) varies as a function of temperature. This response curve is highly nonlinear, with a sharp increase in the equilibrium values over a narrow range of temperatures (electronic supplementary material, figure S7*a*). A sharp transition is defined here as an increment in the fraction of infected humans of more than 0.8 between 18 and 20°C. Approximately one-third of the solution ensemble exhibits this behaviour, with a gradation of more gentle responses for the rest. Typical values for the distribution of this transition value are within the variation of the observed mean temperatures (electronic supplementary material, figure S7*b*,*c*), implying that the system would have crossed this point more than once during the seasonal excursions of temperature, which explains the large increase in the projected cases despite the small trend in temperature.

The above analyses considered a constant population size of 50 000. Similar results are obtained with constant populations ranging from 25 000 to 75 000 (electronic supplementary material, figure S12). Although the population size inside the tea estate is described in the literature as largely constant, we can ask about the potential consequences of a growing population in the surrounding town and rural areas [21] for both the parametrization of our model and the higher malaria prevalence observed in the 1990s. Figure S8 in the electronic supplementary material shows the exponential increase in the rural and urban areas of the town of Kericho for the study period. More human hosts outside the tea estates, and more malaria carriers, could influence transmission dynamics in different ways. They could increase the external force of infection, as the result of workers and their dependants becoming infected outside the tea estate. We ask whether the data provide evidence for such a trend in the first part of the time series by fitting the model with a linear trend in the external force of infection. The fitted slope of this linear trend is not significantly different from zero (electronic supplementary material, figure S9), and the likelihoods of the model are similar to those of our original formulation (electronic supplementary material, figure S10). The parameters obtained when such a trend is included give rise to similar ranges of malaria increases in the 1990s (results not shown). Another possibility, at the opposite end of the mixing spectrum, is that a model that considers a non-zero growth rate inside the tea estate could fit our data better. To address this possibility, we fitted an extra parameter—the human birth rate—and compared it with the death rate (or turnover rate). These two parameters were set equal in the previous analyses to maintain a constant population. When we allow them to differ, there is no evidence for a significant difference between birth and death rates (see electronic supplementary material, figure S11). In particular, this implies that a model with a positive exponential growth rate (such as the one observed outside the tea estates) would fit the data less well than the model with a constant population size. These analyses indicate that the parametrization of our model is robust to these two potential effects of population growth outside the tea estate. They do not mean, however, that a growing population outside the tea estates, or a related increase in the immigration of cases from outside the system, has not acted in the 1990s. These trends belong to a suite of factors that can increase or decrease the change in malaria owing to warmer temperatures projected by our model. We return to population growth in this context in §4.

## 4. Discussion

Our results support a significant role of warmer temperatures in the exacerbation of the disease from the 1970s to the 1990s. The fact that the increases projected by the model were nevertheless typically smaller than those observed in the data is consistent with additional factors other than temperature also being at play. These factors include drug resistance of the parasite to chloroquine (which emerged in Kenya at the beginning of the 1980s [13,15]), land-use patterns (which can influence effective local temperatures [24]), the rise of HIV prevalence [25], increased human movement [13], population growth and the associated deterioration of health services [12]. Neither the quality of health services nor the size of the population of workers and their dependants appears to have changed significantly over the past three decades, and although a considerable fraction of the population travels regularly to lower, more endemic regions, this travel is not new [13,15]. Thus, the tea plantation data provide an opportunity to estimate the magnitude of the effect of warmer temperatures in a more ‘controlled’ setting than in its surrounding regions. There, too, the burden of malaria has increased [21,26]. Our results suggest that warmer temperatures can explain a significant portion of this increase, acting in the same direction, if not synergistically [24], with change in other important factors.

Our results appear robust to different variations in model structure, including the specific description of population immunity and how repeated exposure to the disease affects infectiousness and immunity. This flexibility in the model formulation is important because the representation of immunity in malaria models can take several forms, but also because there is evidence for the existence of acquired functional immunity and asymptomatic infections in other highland areas of Kenya based on the age structure of cases and molecular studies [12,27].

We have considered mean temperatures and not the higher temporal resolution of diurnal variation. One important future direction will be to consider the variation in minimum and maximum temperatures [28–31]. Refinements of our analysis would further benefit from consideration of the human population distribution with altitude, and from measurements of indoor versus outdoor temperatures for this specific location. We have considered here a maximum difference of 5°C as reported for the tea plantation [15]. At lower altitudes in the same region (1430–1580 m above sea level), a maximum difference of 3.2° has been reported [32]. However, a value of 5° is better supported by a higher likelihood of our model for that difference than for lower ones. Detailed temperature measurements every half-hour for six months at another East African highland (in Ethiopia) indicate differences up to 5 and 6°C, between temperatures outdoors (ambient and within vegetation) and indoors (human dwellings with different types of roofs and uses), at 1950 m (J. Cox, A. Tulu & M. J. Bouma 1998, unpublished data; see electronic supplementary material, figure S7). The pioneer studies of Garnham [33] in the Kenyan highlands described larger differences between uninhabited and inhabited huts under certain weather conditions at similar altitudes (fig. 4 in Garnham [33]). In our model, a phenomenological parameter fitted to the data represents implicitly the myriad mechanisms, including behaviour, that can alter the temperature perceived by adult mosquitoes [34]. Thus, the resulting effective difference does not need to simply reflect indoor versus outdoor temperatures. The resulting numbers of adult mosquitoes predicted in our simulations range typically from 0.05 to 0.5 individuals per human and are consistent with empirical observations in these regions [35], even though the model was fitted to malaria cases only, with all other variables in the model, including vector abundances, as ‘hidden’ variables for which time series data were not available. The collection of malaria vector densities in highland areas is especially difficult given their low numbers and the intermittent character of epidemics [28].

Our results suggest that some local transmission was already present in the region in the 1970s and at the beginning of the 1980s. The model incorporates both an external and an internal force of infection to represent, respectively, the importation of infection from lower regions outside the tea estates and the local transmission via the mosquito vector. The internal force of infection becomes more prominent in the 1990s with the increase in temperatures (see electronic supplementary material, figure S6, for a related measure of infection intensity, the EIR, measuring the number of infectious bites per person per year).

The model projections in the 1990s capture the timing (year) of two out of the three major epidemics. This suggests that temperature plays a role in the interannual variability of the disease, and leads to open questions on the relationship between temperature and rainfall anomalies in this region, given the previously described role of rainfall in the interannual variability of malaria in the tea estates [21,22]. The simulations also suggest that any trend in the rainfall data itself (for example in its variability over time) does not produce a significant increase in cases across these decades. This is apparent in the baseline simulations that contain the effect of the observed rainfall patterns but not the temperature trend, and do not show significant increases in cases. This does not preclude, however, an interaction between rainfall and temperature, so that a trend in the variability of the former would only manifest itself under warmer temperatures—a possibility that remains to be examined.

The model is less able to capture precisely the monthly timing of seasonal epidemics, especially for one large peak in 1997 the timing of which is delayed in the simulations. The complexity of the seasonal pattern, with two peaks per year (a main peak following the main rainfall season and an earlier, smaller peak following the short rains) may find an explanation in the complex ecology of the vector(s) [22]. This level of mechanism, especially if two different vectors are involved, cannot be reproduced by our model explicitly, particularly given the ‘anomalous’ timing of the 1997 outbreak and the short length of the record exhibiting epidemics. A better result would have been obtained if we had fitted the model to the whole time series, including the second part when the seasonal patterns become more evident, but this was not the goal of our analysis. We specifically avoided fitting the whole time series because this would have implicitly assumed, *a priori*, that temperature alone was responsible for the observed epidemics. Thus, we do not expect our projections after 1985 to be able to accurately predict the observed patterns; they are instead intended to quantify the magnitude of the increase in cases that temperature can explain on its own.

We have considered different human sub-models to examine the robustness of the results to the structure and representation of acquired immunity (and super-infection; electronic supplementary material). An open question is whether these different representations have important dynamical consequences, in terms of characteristic scales of interannual variability and bifurcation patterns with increasing transmission intensity. For our purposes here, these sub-models, with two levels of susceptibility and infection, provide a representation of the dynamics of human malaria at the population level that goes beyond simpler SIRS formulations but remains sufficiently parsimonious to be coupled to the mosquito component and confronted to population-level time-series data. Future extensions of the human sub-model(s) could incorporate age explicitly [36] in formulations with multiple levels of immunity and infection (parasitaemia [37]). Parameter estimation for such extensions will require additional data at the individual level to complement the population time series. For endemic or ‘stable’ malaria regions, epidemiological models with multiple immunity levels have been developed and parametrized based on age-specific prevalence, levels of parasitaemia and infectivity from epidemiological surveys [37]. For more epidemic or ‘unstable’ regions, population-level time series provide key information on the more dynamic behaviour of cases over time, especially in the interannual variability in the size of epidemics [38]. Ongoing efforts seek to combine epidemiological data at these different levels of organization for inference purposes of more complex transmission models. Although the expectation would be that more detailed representations of acquired immunity will not be critical at the lower end of the transmission spectrum, it will be valuable to confirm this and to develop models that can be applied across broad ranges of transmission intensities.

We have considered a constant population with turnover. The population of Unilever's (formerly Brooke Bond's) estate has been reported to have remained largely unchanged over the years [15,16,39]. There has been, however, significant growth of the population in the nearby town (around 4–5% annually; electronic supplementary material, figure S8), and the question of the possible effect of such growth on malaria prevalence in the tea estates arises. We have shown that, despite such growth, the malaria data we have used to fit the models do not support an increase in either the external force of infection or the local population within the tea estates (in agreement with the literature) for the first half of the time series (electronic supplementary material, §11). It is important to note that these analyses do not address (and are not meant to address) whether a rise in the external force of infection is at play later on in the 1990s, but rather whether the estimated model parameters are robust to relaxing the assumption of a constant force of infection and a constant population. As we have already emphasized, we recognize that drivers other than temperature are likely to have influenced malaria prevalence, and that these would act to modify the estimated increase owing to temperature alone. Our study was specifically designed to estimate the effect of temperature in isolation from other trends. This is because the meaningful consideration of such trends would require additional time-series data (for example, on levels of drug resistance, HIV/AIDS and human movement), and even then it would be difficult to statistically differentiate the respective effects of multiple trends. Most of these trends would add, however, to the increase in cases projected for warmer temperatures. In particular, a rising external force of infection is likely with patterns of increased movement and growing surrounding populations, and this would act to further increase malaria prevalence beyond the rise generated by temperature. In this regard, the expansion of the human population into valleys within the highlands that can act as a reservoir for malaria and provide a source of transmission for higher, uphill areas [21] is especially relevant.

The consideration and consequences of a growing population in surrounding areas of the tea plantation through the modification of the internal force of infection is less evident for several reasons. First, population growth in models for vector-transmitted diseases (with a single host), such as ours, decreases the force of infection within the system: this is because a larger number of hosts do not change the number of vectors, and this leads to a decrease in both the fraction of mosquitoes per human and the force of infection, which is a function of this ratio. Thus, a larger population acts in this way to effectively decrease transmission intensity in models with frequency-dependent transmission (electronic supplementary material, §11). A growing local population *per se* cannot explain an increase in prevalence simply as the result of the transmission dynamics, unless the models were to incorporate indirect (and potentially important) mechanisms by which higher populations and/or densities of humans lead to more mosquitoes, especially in anthropophilic vectors such as *Anopheles gambiae*. This brings us to our second point. Population growth can act in more complex ways than the dilution effect described above, including the opposite direction, through heterogeneous biting by the vector [40] and mechanisms associated with human settlement that increase breeding sites for the vectors or stress the capacity of declining health services. These mechanisms are difficult to parametrize without specific empirical studies, including the effects of human densities versus abundances, on the vector to human ratio. Third, a single, well-mixed population with exponential growth would provide a poor representation of the system at large, which is effectively composed of two subpopulations: the tea plantation and the surrounding areas. The tea estates have designated areas with company-built standard housing for staff and family only. Clusters of these houses are found over the tea plantation (in a strip of around 25 km that is on one side flanked by another tea plantation), and transmission takes place primarily at night when the population of the tea estates is spatially segregated from the surrounding rural population. Typical distances to the suburban areas of the town of Kericho exceed those that *An. gambiae* would typically travel when searching for blood meals [41]. Therefore, the populations inside and outside the tea plantation are not well mixed from the perspective of malaria transmission, and the degree of mixing via the vector is unlikely to have biased our results. In addition, the two subpopulations exhibit different demography (and probably different treatment levels). All these factors indicate that an extension of our model (without population growth and only one subpopulation) would require the explicit consideration of the two subpopulations, and its parametrization would require the consideration of at least the additional data on the number of cases outside the tea plantation. Importantly, however, population growth could act to either increase or decrease the effect of warmer temperatures on malaria prevalence in other highland locations, depending on the balance of the opposite forces described here, and this should be considered in extrapolations of our work beyond the tea plantations, given the pronounced demographic expansion in highland regions of East Africa. Rising population (densities) could also play a role in the intensity of transmission in very sparsely populated areas, such as deserts, and particularly when vectors are poor (zoophilic), and the animal to human feeding ratio could change, resulting in more bites on humans [42].

An increase in transmission intensity, regardless of the underlying drivers, does not preclude the potential effectiveness of control measures in these regions. On the contrary, it underscores the importance of such measures, and of studies evaluating the extent to which trends in disease risk resulting from warmer temperatures will increase the need and costs of intervention [43]. Control efforts including insecticide spraying have risen in Kenyan highlands (including the tea estates) in the last decade, and several recent studies demonstrate the potential effectiveness of such efforts in ‘unstable’ transmission settings, where transmission intensity decreases significantly during the dry season. For example, the addition of mosquito larval control with microbial larvicides to insecticide-treated nets was shown to confer significant additional protection against malaria parasites in highland regions [44]. The last 2 years in the time series analysed here exhibits a decrease in cases that was explained by a change in drug treatment by Shanks *et al.* [15]; the epidemiological dynamics themselves may also have contributed to this pattern given the decrease present in our model projections for that period (figure 3*a*). The combination of regular, widespread indoor residual spraying with long-lasting insecticides and the use of ACT as first-line antimalarial drug treatment reduced and possibly interrupted local malaria transmission in two adjacent highland areas from April 2007 to March 2008, following a series of interventions started in 2005 by the Ministry of Health of Kenya [45]. As pointed out by the authors [45], however, the sustained elimination of malaria will require the reduction and eventual elimination of malaria in surrounding, more endemic areas, with the possibility of the development of resistance in the vector and/or parasite.

Finally, the general approach we have used here should be applicable to retrospective records in other highland regions for which both malaria cases and meteorological variables exist. It would be informative to compare the conclusions of this dynamical approach across different regions, particularly if data on other drivers such as drug resistance were also available. In the meantime, this study already underscores the nonlinear response of malaria dynamics to increases in temperature, with small temperature differences amplified in the disease response.
